# Transfer Entropy Granger Causality between News Indices and Stock Markets in U.S. and Latin America during the COVID-19 Pandemic

**DOI:** 10.3390/e24101420

**Published:** 2022-10-05

**Authors:** Semei Coronado, Jose N. Martinez, Victor Gualajara, Omar Rojas

**Affiliations:** 1 Palomar College, San Marcos, CA 92069, USA; 2 Accounting, Finance and Economics Department, California State University, Dominguez Hills, Carson, CA 90747, USA; 3 Departamento de Métodos Cuantitativos, Centro Universitario de Ciencias Económico Administrativas, Universidad de Guadalajara, Guadalajara 44100, Jalisco, Mexico; 4 Facultad de Ciencias Económicas y Empresariales, Universidad Panamericana, Zapopan 45010, Jalisco, Mexico; 5 Faculty of Economics and Business, Universitas Airlangga, Surabaya 60286, East Java, Indonesia

**Keywords:** MODWT, Granger causality, transfer entropy

## Abstract

The relationship between three different groups of COVID-19 news series and stock market volatility for several Latin American countries and the U.S. are analyzed. To confirm the relationship between these series, a maximal overlap discrete wavelet transform (MODWT) was applied to determine the specific periods wherein each pair of series is significantly correlated. To determine if the news series cause Latin American stock markets’ volatility, a one-sided Granger causality test based on transfer entropy (GC-TE) was applied. The results confirm that the U.S. and Latin American stock markets react differently to COVID-19 news. Some of the most statistically significant results were obtained from the reporting case index (RCI), A-COVID index, and uncertainty index, in that order, which are statistically significant for the majority of Latin American stock markets. Altogether, the results suggest these COVID-19 news indices could be used to forecast stock market volatility in the U.S. and Latin America.

## 1. Introduction

The world pandemic, declared by the World Health Organization (WHO) on 11 March, 2020 [1], caused by the coronavirus (COVID-19), represents an unprecedented shock to the world economy. Starting in Wuhan, China, in December 2019, the shock spread worldwide, but its economic consequences have been particularly strong for Latin American countries (Economic Commission for Latin America and the Caribbean, 2020). This pandemic has been characterized by a climate of panic and uncertainty that has shocked world financial markets. At the same time, this uncertainty has forced some economic agents to leave the stock market entirely, which can create changes in volatility [2,3].

For example, the Standard and Poor’s 500 index (SP), which measures the performance of 500 major companies in the U.S. stock market, lost almost 30% of its value since the pandemic’s beginning. Between March 6 and 18, 2020, the index fell by nearly 15% [4]. Government intervention, like the implementation of the Coronavirus Aid, Relief, and Economic Security (CARES) Act, helped stabilize the financial market in the U.S. [5,6]. In Latin America, countries tend to lack the financial resources to confront the impacts of the pandemic on their economies and financial markets, which lead to reductions in economic growth and capital investments. However, it is not clear how the financial markets in these countries react to such types of reductions, but what is known is that the stock markets somehow responded to the pandemic [7]. Financial markets reacted in a specific way due to the heightened level of uncertainty brought by the pandemic, and that led some researchers to try to understand such a reaction, specifically in terms of volatility, the measure of which is useful to obtain better financial returns using portfolio diversification [8].

Different studies have tried to understand the volatility of financial markets and have related it to other economic, financial, and other types of variables during the pandemic [9,10,11]. Additionally, some studies have analyzed the relationship between COVID-19 news, like the Infectious Disease Equity Market Volatility Tracker (EMV-ID) series [12] and different economic and financial variables [13,14,15,16].

Other real-time news indices related to COVID-19, like Ravenpack’s indices, have been associated with different financial and economic variables [17,18,19]. However, there are other news indices that have not been studied much for Latin American stock markets, like the COVID-19 reporting case index (RCI), the reporting death index (RDI), and the global fear index (GFI), created by [19]. Another one is the COVID-19-induced uncertainty stock tracker composite index (ciustk.cmp). This index combines two indices, an index that has global news related to COVID-19 (ciustk.news) and the index of uncertainty due to global economic indicators (ciustk.mac), which contains information on the price of oil, gold, raw materials, exchange rates, and stock prices [20]. The last set of global news indices includes the aggregate index of COVID-19, medical index, travel index, uncertainty index, vaccine index, and COVID index [21].

This study aims to analyze different types of COVID-19 news indices through time and determine how they might affect the volatility of stock markets in the U.S. (SP), Mexico (MEXBOL), Colombia (COLCAP), Brazil (BOVESPA), Peru (IGBVL), Argentina (MERVAL), and Chile (IPSA) (These series were obtained from Bloomberg). This paper applies the [22] maximal overlap discrete wavelet transform (MODWT), which can be applied to different types of series to calculate the wavelet multiple correlations, which help determine the specific periods where each pair of series is significantly correlated [23,24,25].

This study contributes to the literature on the co-movement of these new stock market news indices in several ways. First, to the best of our knowledge, these news series have not been studied to analyze their co-movement with some Latin-American stock markets. Second, the SP stock market of the United States is used as a developed market, and the results are compared with those of the Latin American market. Third, the evidence shows that these series have been used with other types of economic and/or financial series from other markets to measure the effect through autoregressive vectors [26], the asymmetric impact [27] utilizing an NARDL model. Therefore, MODWT is a model that can be used to determine the correlation of the series at different scales, transforming a signal into multiple wavelets and scale coefficients compared to the discrete wavelet transform (DWT), which is orthogonal, requires decomposing the series orthogonally with different frequencies, and is not manageable for any sample size in the series in signs that fits this study [28].

However, as is well known, correlation does not imply causality [29,30]. Therefore, to determine if the news series cause stock market volatility and can be used to forecast the Latin American stock markets’ volatility, we apply a new one-sided Granger causality test based on transfer entropy (GC-TE) [31]. The Granger causality test is based on an autoregressive model, which has expanded its construction in neural networks and state-space [32]. However, entropy transfer is derived from information theory, and it does not require assumptions of cointegration between the series [33]. It can quantify the amount of information transferred between two random processes, which can be related as a measure of causality [34]. Therefore, entropy can be seen as a measure of uncertainty, which can be seen as a measure of volatility; the greater the volatility (uncertainty), the greater the randomness in the transmitted information, which can be seen as Granger-type causality [35]. This new test is based on the first-order Taylor expansion of entropy, shown to be asymptotically normally distributed [31].

Several factors motivated the analysis of the impact of these news indices on different Latin American and U.S. stock markets. First, financial markets’ reactions might depend on each country’s cultural differences, which derive from the collective behavior of individual investors and their decision-making, which influences how they interpret and react to pandemic information as it arrives [36]. Second, investor sentiment also played an important role during the pandemic; their relative optimism or pessimism can determine their actions and cause investors’ confidence to shift market volatility downwards or upwards [37]. Furthermore, these news indices might cause some investors not to pay attention to the market as bad news arrives, which would change the volatility because they prefer to do nothing instead of making optimal decisions in their portfolios [38].

This paper is organized into four sections: Section 2 presents the materials and methods; Section 3 discusses the empirical results; Section 4 presents the conclusions.

## 2. Materials and Methods

This study employs the MODWT and GC-TE tests to analyze COVID-19 news indices and stock markets’ volatility. The MODWT is used because the different stock markets in Latin America usually react to different types of news, announcements, or financial events that occur in the U.S. stock market [39,40,41]. Furthermore, investors must make decisions considering different time horizons at a different time or frequency [42]. Furthermore, the MODWT can calculate the variance of the wavelets and their coefficients at different scales, and the variance estimator is asymptotically more efficient [43,44]. Finally, if the financial series are non-linear for different reasons, calculating a linear correlation or linear causality between them could result in non-significant results [45]. For this last reason, GC-TE provides a good fit due to the nonlinear relationship that could exist between the variables [31,46].

The MODWT can be applied to any *n*-sized sample; it is invariant to circular shifting in the analyzed time series. It can analyze variance based on wavelet and scaling coefficients [22,47]. The MODWT unbiased estimator of the wavelet correlation for scale $\lambda_{j}$ between two series, *X* and *Y,* can be described as

(1) $${\tilde{\rho }}_{XY}=\frac{cov\left({\tilde{W}}_{X,j,t},{\tilde{W}}_{Y,j,t}\right)}{\sqrt{var\left\{{\tilde{W}}_{X,j,t}\right\}var\left\{{\tilde{W}}_{Y,j,t}\right\}}}=\frac{{\tilde{\gamma }}_{XY}\left(\lambda_{j}\right)}{{\tilde{\sigma }}_{X}\left(\lambda_{j}\right){\tilde{\sigma }}_{Y}\left(\lambda_{j}\right)}$$

According to Polanco-Martínez and Abadie (2016), ${\tilde{\gamma }}_{XY}\left(\lambda_{j}\right)$ is an unbiased estimator of the wavelet covariance between wavelet coefficients ${\tilde{W}}_{X,j,t}{\tilde{,W}}_{Y,j,t\;},{\tilde{\sigma }}_{X}\left(\lambda_{j}\right)$, and ${\tilde{\sigma }}_{Y}\left(\lambda_{j}\right)$ are unbiased estimators of the wavelet variances for *X* and *Y*, respectively, associated with scale *j*. The following is an unbiased wavelet variance estimator based on the MODWT [43].

(2) $${\tilde{\sigma }}_{X}^{2}\left(\lambda_{j}\right)=\frac{1}{{\tilde{N}}_{j}}\overset{N-1}{\underset{t=L_{j}-1}{\sum }}{\tilde{W}}_{j,t}^{2}$$

*j* is the wavelet coefficient level associated with changes at the practical scale $\lambda_{j}=2^{j-1}$ within the frequency interval $\left[\frac{1}{2^{j+1}},\frac{1}{2^{j}}\right]$ for scale levels 1≤j≤1; *J* is the maximum decomposition at a level by $log_{2}\left(N\right)$; *N* is the length of data; $\left\{{\tilde{W}}_{j,t}\right\}$ are the *j-th* level MODWT wavelet coefficients for the time series *X*; $L_{j}=\left(2^{j}-1\right)\left(L-1\right)+1$ is the length of the scale $\lambda_{j}$ wavelet filter; *L* is the length of Daubechies least asymmetric (LA) wavelet filter, and ${\tilde{N}}_{j}=N-L_{j}+1$ is the number of coefficients not affected by the boundary.

The Granger causality test based on the transfer entropy (GC-TE) is a modification of the Granger causality nonparametric test by [48]. This new modification is based on a first-order Taylor expansion of the transfer entropy, which is shown to be asymptotically normally distributed [31]. Transfer entropy was initially used to measure asymmetric information exchange in bivariate systems. With appropriate conditional densities, transfer entropy can measure the transfer of information from one variable to another. Granger causality is calculated as follow:

(3) $$T_{n}^{\prime }\left(h\right)=\frac{\left(n-1\right)}{n\left(n-2\right)}\overset{n}{\underset{i=j}{\sum }}\left[\left(\hat{f}\left(X_{i},Y_{i},Z_{i}\right){\hat{f}}_{X,Y}\left(X_{i},Y_{i}\right){\hat{f}}_{Y,Z}\left(Y_{i},Z_{i}\right)\right)\frac{1}{\hat{\upsilon }\left(X_{i},Y_{i},Z_{i}\right)}\right]$$

where $\hat{\upsilon }=\left(X_{i},Y_{i},Z_{i}\right)={\hat{f}}_{X,Y}\left(X,Y\right){\hat{f}}_{Y,Z}\left(Y,Z\right)$ The bandwidth $h^{*}=4.8n^{\frac{-2}{7}}$ for GARCH or ARCH process has a standard normal distribution as its limiting distribution. To know more about GC-TE, see [31].

There are three groups of news indices with different periods considered in this document—the first group of indices [49] from 11 February 2020 to 29 October 2021 (448 observations); the second group of indices [20] from 3 March 2020 to 27 January 2022 (497 observations); the third group of indices [21] from 1 January 2020 to 28 April 21 (345 observations). To consider the three different periods, the EMV-ID index and the stock markets were matched according to the specific periods. The series were transformed into growth rates $y_{t}=100\left(\ln\left(x_{t}\right)-\ln\left(x_{t-1}\right)\right)$. Table 1 shows the descriptive statistics of $y_{t}$. The statistics in all samples are consistent with the typical stylized facts of financial time series (see [50]). The Jarque–Bera test [51] in samples is right-skewed and leptokurtic. To determine if the series is stationary, the residual augmented least square (RALS) test of [52] was applied, and its results was significant. In addition, the BDS test [53] was applied, and its results were significant. These results indicate that the series have non-linear behaviors.

News indices are considered volatility, so the volatility of each of the financial markets was obtained using a GARCH model, once the logarithmic rate was determined.

The MODWT used by default is the least asymmetrical Daubechies wavelet filter of length 8 [42]. Ref. [47] showed that the maximum decomposition level *J* = 8; the scale in days is calculated as $\lambda_{j}=2^{j-1}$; the equivalent periods is $[2^{j},2^{j+1}]\Delta t$ with *j* = 1, 2, 3, 4, 5, 6, 7, 8, and Δt in days. Thus, wavelet coefficients are associated with days periods of (2, 4], (4, 8], (8, 16], (16, 32], (32, 64], (64, 128], (128, 256], and (256, 512] in days, respectively.

The GC-TE test is applied to the specific periods used in the MODWT test, a skewed *t*-distribution, and bandwidth of *h* = 0.84 for the first group of series, *h* = 0.82 and *h* = 0.90 for groups two and three, respectively.

## 3. Results and Discussion

**Figure 1:**
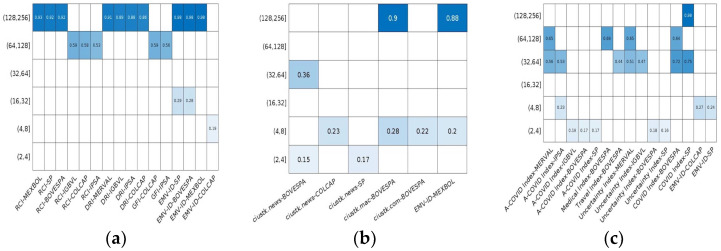
Wavelet correlation between news indices and different stock markets from Latin America. Note: (**a**) correspond to group 1, (**b**) correspond to group 2, and (**c**) correspond to group 3. The color scale shows the strength of the wavelet correlation coefficients, from weak correlations in light blue to strong ones in blue, and white indicates no correlation. The correlation was calculated with W2CWM2C, an R package, and Matlab.

Figure 1 shows the correlation coefficients for each pair of series (stock market and news indices from three different groups) using MODWT. The results indicate that correlation was found between some pairs of series between (2, 4] days and up to (128, 256] days. For some series, a low correlation was found between the first days and the first two weeks; later, it disappeared, and it reappeared with a moderately low correlation between 0.20 and 0.36; there is a high quarterly (64, 128] and semiannual (128, 256] correlation above 0.95 in some cases.

The correlation of the different indices varies across countries; it is lower in the first weeks of the year, reaching the highest correlation at the end of the year This can be attributed to the application of different public, financial, or economic policies to counteract the effects of the pandemic. For example, once the news of the first COVID case was reported, BOVESPA fell by −7.26% and MEXBOL by −4.59%. By the end of April 2020, the stock markets continued to decline, but the volatility was higher [54]. Furthermore, news about vaccination is correlated with the stock market, since the MERVAL, BOVESPA, and IPSA increased their volatility. At the same time, the IPC decreased because countries were acquiring different vaccines for COVID-19 by 2022 [55]. For its part, the EMV-ID has a low correlation with the SP in the first days or up to a month later. These results are consistent with [13,14,15,16] regarding the correlation between the EMV-ID and the SP.

The BOVESPA stock market is frequently correlated with different news indices. On the other hand, the MEXBOL shows a high correlation with the EMV-ID. The news indices of groups 2 and 3 are the ones that present a more significant correlation with some Latin American financial markets, without ceasing to affect the SP of the U.S.

These results show a strong correlation of the COVID-19 news and stock markets between a month and up to a year, which implies that investors could optimize their portfolios in advance.

In addition, governments could establish economic policies to stabilize the market in the face of what happens in the U.S.

Using only the pairs of series in Figure 1 that were statistically significantly correlated, Table 2 presents the results of the GC-TE test in a unidirectional way. These results indicate that some of these news indices have a strong correlation with the different Latin American stock markets for about six months and show Granger causality, which suggests that they can be used to forecast stock market volatility. With that in mind, the EMV-ID index continues to be statistically significant for stock markets’ volatility using different periods. However, some of the most statistically significant indices are the RCI, A-COVID Index, and uncertainty index, in that order, which are statistically significant for the majority of stock markets. Regarding the results for the ciustk.news, ciustk.mac, ciustk.com indices, and uncertainty index, they are aligned with [56,57], who discovered a connection between some macro-level news and price volatility. The travel index, medical index, the COVID index, and the A-COVID index are statistically significant for Latin American stock markets; these results present evidence that Latin American markets react differently to international news than American markets. Altogether, the results suggest that these COVID-19 news indices can be used to forecast stock market volatility in the U.S. and Latin America.

## 4. Conclusions

The heightened level of uncertainty in financial markets brought by the COVID-19 pandemic has led researchers to try to understand the relationship between stock market volatility and COVID-19 news series. This study analyzed different types of COVID-19 news series through time to determine how they might affect the volatility of stock markets in several Latin American countries and the U.S.

This document applied wavelets through MODWT to determine the correlation to each pair of series and GC-TE to determine the instantaneous, one-way Granger causality using entropy between the news series and the stock market series in the U.S. and Latin America.

Overall, COVID-19 news impacted U.S. financial markets differently than emerging markets. Furthermore, the EMV-ID index continues to be statistically significant for some countries’ stock market. However, some of the most statistically significant indices are for the RCI, A-COVID index, and uncertainty index, in that order, which are statistically significant for the majority of Latin American stock markets. In particular, the RCI was only statistically correlated with the MEXBOL. These findings indicate that, as the information arrives at the Latin American stock markets, the Indices are linked with that until after 128 days. In terms of causality, the one-sided Granger causality tests based on the transfer entropy confirm that the U.S. and Latin American markets react differently to COVID-19 news. Regarding policy implications, the principal results suggest that investors in Latin American markets tend to pay more attention to news that occurs in the U.S.

Altogether, the results suggest these COVID-19 news indices can be used to forecast stock market volatility in the U.S. and Latin America. In addition, these results show the continuing dependence of Latin American financial markets on the U.S. In terms of future research, the analysis presented in this document can be expanded to include European and/or Asian markets.

## Figures and Tables

**Table 1:** Descriptive Statistic of $y_{t}$.

Group	Variable	Mean	Median	Min	Max	Variance	Standard Deviation	Skewness	Kurtosis	Jarque–Bera	RALS	BDS
Group 1	RCI	−0.09	−0.33	−132.15	183.71	306.17	17.50	1.36	44.02	31,000	−9.24	9.62
	RDI	−0.03	−0.18	−146.03	121.87	179.75	13.41	−1.35	58.10	57,000	−19.99	13.00
	GFI	−0.06	−0.36	−125.11	166.35	157.13	12.54	2.72	94.48	160,000	−6.40	8.88
	EMV–ID	0.35	−3.22	−275.86	294.28	3625.80	60.21	0.02	5.59	125	−11.11	7.50
	SP	0.01	0.03	−2.22	1.59	0.11	0.33	−1.60	16.34	3504	−6.23	13.00
	MEXBOL	0.01	0.00	−1.27	0.91	0.06	0.25	−0.55	6.22	216	−6.44	10.49
	COLCAP	0.01	0.00	−3.85	7.28	0.38	0.62	2.94	55.28	52,000	−12.22	9.68
	BOVESPA	0.00	0.02	−2.49	1.37	0.10	0.32	−2.03	19.04	5096	−8.15	13.26
	IGBVL	0.01	0.01	−31.72	32.03	4.67	2.16	0.20	212.77	820,000	−35.46	5.74
	IPSA	0.00	0.00	−65.55	67.28	20.02	4.47	0.57	218.52	870,000	−61.48	5.74
	MERVAL	0.02	0.02	−15.36	15.19	1.35	1.16	−0.36	135.20	330,000	−13.56	6.53
Group 2	ciustk.news	−0.02	−0.11	−123.23	103.48	555.26	23.56	0.11	6.54	260	−26.19	6.31
	ciustk.mac	−2.02	1.13	−333.53	86.65	779.07	27.91	−10.22	121.51	300,000	−2.72	14.86
	ciustk.com	−0.06	0.28	−89.10	181.80	172.09	13.12	3.18	91.74	160,000	−11.46	11.34
	EMV–ID	−0.05	−2.97	−275.86	294.28	4192.56	64.75	−0.06	5.29	109	−12.22	8.76
	SP	0.01	0.04	−3.94	2.71	0.15	0.38	−3.50	44.56	37,000	−5.21	13.26
	MEXBOL	0.01	0.01	−2.54	1.80	0.05	0.23	−2.19	40.78	30,000	−7.88	11.49
	COLCAP	0.01	0.00	−3.85	5.30	0.32	0.56	0.89	32.56	18,000	−13.44	9.34
	BOVESPA	0.00	0.02	−2.46	1.79	0.10	0.32	−1.74	20.08	6277	−10.79	13.31
	IGBVL	0.01	0.01	−31.74	32.04	4.21	2.05	0.20	235.87	1,100,000	−37.05	6.05
	IPSA	0.00	0.00	−65.57	67.29	18.06	4.25	0.60	242.02	1,200,000	−63.21	6.05
	MERVAL	0.02	0.02	−15.36	15.19	1.31	1.15	−0.81	131.46	340,000	−14.33	6.96
Group 3	A–COVID Index	0.27	0.05	−133.93	148.16	533.49	23.10	0.38	22.30	5346	−9.97	8.09
	Medical Index	0.21	0.27	−216.02	225.05	695.63	26.37	0.41	39.30	19,000	−38.55	7.96
	Travel Index	−0.06	−0.45	−317.50	297.07	2868.68	53.56	0.05	12.54	1303	−11.29	8.04
	Uncertainty Index	−0.04	−0.67	−106.08	120.38	428.86	20.71	0.40	9.54	623	−8.67	8.71
	vaccine index	0.29	0.34	−398.84	412.21	4462.45	66.80	0.21	15.12	2107	−14.15	8.43
	COVID Index	0.13	0.05	−539.44	549.61	2850.90	53.39	0.28	78.95	83,000	−16.15	7.78
	EMV–ID	3.91	−2.01	−275.86	294.28	3060.36	55.32	0.21	6.48	176	−7.45	3.34
	SP	0.02	0.04	−2.19	1.57	0.13	0.36	−1.40	13.74	1765	−5.22	11.41
	MEXBOL	0.01	0.00	−1.27	1.30	0.08	0.28	−0.23	6.22	152	−7.40	9.62
	COLCAP	0.00	0.00	−3.85	7.22	0.47	0.68	2.79	47.51	29,000	−11.14	8.40
	BOVESPA	0.01	0.03	−2.49	1.68	0.13	0.36	−1.59	16.54	2772	−7.69	11.87
	IGBVL	0.00	0.01	−31.74	32.04	6.04	2.46	0.18	166.03	380,000	−40.88	5.02
	IPSA	0.01	0.01	−65.55	67.28	25.98	5.10	0.50	168.74	390,000	−57.32	5.02
	MERVAL	0.01	0.01	−14.41	14.41	1.50	1.23	−0.09	111.93	170,000	−12.06	3.15

Note: The results of the Jarque–Bera, RALS, and BDS tests were significant at 1%; The results for BDS shows for m(5) and two standard deviations; However, the BDS test was significant for different epsilons and standard deviations.

**Table 2:** GC-TE test unidirectional for pairwise.

RCI→MEXBOL	2.32 ***	ciustk.news→BOVESPA	5.66 ***	A-COVID Index→MERVAL	3.70 ***
RCI→SP	2.27 **	ciustk.news→COLCAP	4.85 ***	A-COVID Index→IPSA	4.01 ***
RCI→BOVESPA	2.55 **	ciustk.news→SP	6.31 ***	A-COVID index→IGBVL	5.15 ***
RCI→IGBVL	2.17 **	ciustk.mac→BOVESPA	2.32 **	A-COVID index→BOVESPA	4.93 ***
RCI→COLCAP	2.58 **	ciustk.com→BOVESPA	4.41 ***	A-COVID index→SP	3.39 ***
RCI→IPSA	2.29 **	EMV-ID→MEXBOL	5.84 ***	Medical index→BOVESPA	3.78 ***
RDI→MERVAL	2.09 **			Travel index→BOVESPA	1.61 *
RDI→IGBVL	0.90			Uncertainty index→MERVAL	3.14 ***
RDI→IPSA	1.21			Uncertainty index→IGBVL	6.03 ***
RDI→COLCAP	2.11 **			Uncertainty index→BOVESPA	6.00 ***
GFI→COLCAP	1.95 *			Uncertainty index→SP	4.64 ***
GFI→IPSA	1.90 *			COVID index→BOVESPA	4.12 ***
EMV-ID→SP	4.63 ***			COVID index→SP	6.46 ***
EMV-ID→BOVESPA	3.55 ***			EMV-ID→COLCAP	5.70 ***
EMV-ID→MEXBOL	4.64 ***			EMV-ID→SP	4.95 ***
EMV-ID→COLCAP	2.19 **				

Note: The asterisks indicate significance at the 10% (*), 5% (**), and 1% (***); The calculations were obtained using MATLAB.

## Data Availability

Not applicable.
